# Lateral Extensional Mode Piezoelectric ZnO-on-Nickel RF MEMS Resonators for Back-End-of-Line Integration

**DOI:** 10.3390/mi14051089

**Published:** 2023-05-22

**Authors:** Adnan Zaman, Abdulrahman Alsolami, Mian Wei, Ivan Rivera, Masoud Baghelani, Jing Wang

**Affiliations:** 1 King Abdulaziz City for Science and Technology, Riyadh 11442, Saudi Arabia; azaman@kacst.edu.sa; 2 Department of Electrical Engineering, College of Engineering, University of South Florida, Tampa, FL 33620, USA; 3 Electrical & Computer Engineering Department, College of Engineering, University of Alberta, Edmonton, AB T6G 2R3, Canada

**Keywords:** electroplated nickel, lateral extensional contour mode, piezoelectric transducer, back-end-of-line integration

## Abstract

High motional resistance and incompatibility with post-CMOS fabrication due to thermal budget constraints are imperative issues associated with the back-end-of-line integration of lateral extensional vibrating micromechanical resonators. This paper presents piezoelectric ZnO-on-nickel resonators as a viable means for mitigating both of the issues. Lateral extensional mode resonators equipped with thin-film piezoelectric transducers can exhibit much lower motional impedances than their capacitive counterparts due to piezo-transducers’ higher electromechanical coupling coefficients. Meanwhile, the employment of electroplated nickel as the structural material allows the process temperature to be kept lower than 300 °C, which is low enough for the post-CMOS resonator fabrication. In this work, various geometrical rectangular and square plates resonators are investigated. Moreover, parallel combination of several resonators into a mechanically coupled array was explored as a systematic approach to lower motional resistance from ~1 kΩs to 0.562 kΩs. Higher order modes were investigated for achieving higher resonance frequencies up to 1.57 GHz. Local annealing by Joule heating was also exploited for quality factor improvement after device fabrication by ~2× enhancement and breaking the record of MEMS electroplated nickel resonators in lowering insertion loss to ~10 dB.

## 1. Introduction

Recent rapid advances in the field of radio frequency microelectromechanical system (RF MEMS) technology have spurred new vigor for multi-channel, multi-standard monolithic transceivers. Among all the RF MEMS devices, on-chip vibrating micromechanical resonators have attracted a great deal of interest due to their high quality, small size and virtually zero power consumption. These great attributes, if associated with post-IC integration compatibility and a low motional resistance for impedance matching with circuits and modules at RF front ends, could position the vibrating MEMS lateral-extensional mode resonators as the best alternatives for the bulky off-chip quartz crystals and ceramic resonators [1,2,3,4,5,6].

There are several viable strategies for reducing the motional resistances of lateral-extensional mode resonators. Capacitive resonators have intrinsic high motional resistances because of relatively weak electrostatic transduction. Thus, low motional resistances can only be achieved by either increasing the DC bias voltage well beyond the typical IC operation voltage, thus needing special circuits such as a charge pump, or by reducing the electrode-to-resonator capacitive transducer nanogaps, which makes it fairly challenging to achieve high-yield volume production. Even through these techniques, the lowest achievable motional resistances are still on the orders of kΩs that are too large to be efficiently coupled with circuits at the RF front-ends [7,8,9,10,11].

Piezoelectric transduction mechanisms have been exploited for lowering a resonator’s motional resistance [12,13,14,15,16,17]. By taking full advantage of the strong piezoelectric coefficient of sputtered c-axis-aligned ZnO thin films, this work investigates ZnO-on-nickel lateral-extensional mode resonators that are capable of realizing high electromechanical coupling (kt^2^) and low motional impedances. Another critical issue associated with such MEMS resonators is IC compatibility because of the required low thermal budget for post-CMOS back-end-of-line integration [14]. Due to the low deposition rates, the thickness of RF magnetron-sputtered ZnO films is often limited to a couple of micrometers or less. Moreover, the fabrication tolerance and yield of thin-film ZnO lateral-extensional mode resonators are affected by the releasing step, as the residual stress causes suspended ZnO structural layers to buckle or even fracture [13,14,15,16,17,18,19]. To circumvent these issues, this work investigates the employment of a nickel thin film, which is deposited by a low-temperature electroplating process, as the acoustic structural material for the vibrating micromechanical resonator body.

Another key issue for vibrating micromechanical resonators is related to their limited frequency range. Scaling down of the characteristic lateral dimensions of is a traditional method for increasing the resonance frequency, which causes environmental susceptibility and more importantly quality factor reduction due to scaling-induced extra losses. This work explores higher order modes for achieving GHz frequencies without excessive size scaling. In addition, the motional resistance can be further reduced by mechanically coupling resonators into an array. It is observed that a localized annealing via Joule heating can be adopted to systematically improve the achievable quality factor. Lateral-extensional, rectangular-plate resonators and resonator arrays made of stacked ZnO piezoelectric transducers on nickel structure are explored, and are amenable to batch fabrication by a post-IC-compatible low-temperature process, while the best achievable Q factors can be further improved by localized annealing [20,21,22]. Lateral-extensional mode ZnO-on-nickel rectangular-plate resonators are designed, fabricated and characterized. The effects of the pitch size and length of interdigitated transducer (IDT) electrodes on the resonance frequency, motional resistance and Q factor are examined experimentally. Furthermore, arrays of parallelly coupled resonators are demonstrated, and result in significantly lower effective motional resistance without affecting the Q factor.

## 2. Device Structure and Operation

The investigated resonator device consists of a rectangular plate operating in its lateral width-extensional mode, which is made of a 750-nm-thick ZnO piezo-transducer layer sandwiched between two metal electrode layers, which are all stacked on top of a much thicker (>3 µm) electroplated nickel structural layer to form the resonator body for storing acoustic energy with fairly low losses among low-temperature materials.

**Figure 1:**
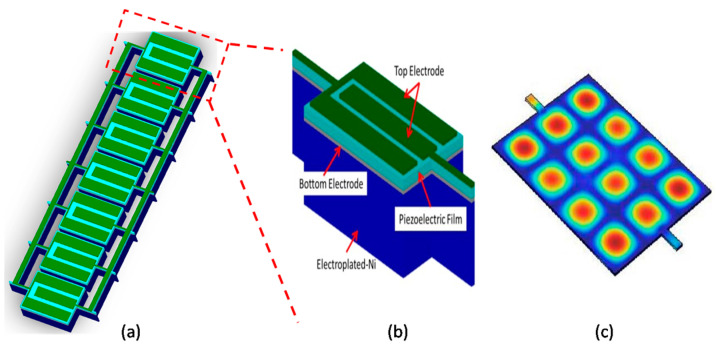
(**a**) Schematic diagram of a lateral-extensional mode ZnO piezoelectric-on-nickel resonator; (**b**) simulated modal response of the mentioned resonator; (**c**) simulated mode shape of a lateral-extensional mode using COMSOL Multiphysics.

Figure 1 illustrates the device structure and modal operation in its third width-extensional mode rendered with a strain field pattern. The width-extensional mode frequency is given by:

(1) $$f_{n}=\frac{n}{2W}\sqrt{\frac{E}{\rho }}$$

where *E* and *ρ* represent the equivalent Young’s modulus and the density of the stacked ZnO-on-nickel structural materials, *W* is the width of the rectangular plate resonator body and *n* is the total number of the interdigital (IDT) electrode fingers.

## 3. Experimental Results

**Figure 2:**
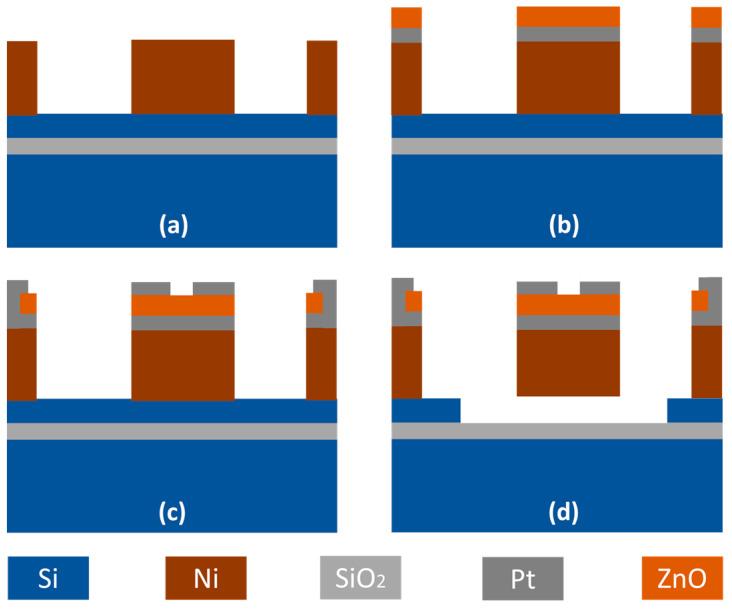
Fabrication process flow for ZnO-on-nickel lateral extensional resonators. (**a**) Depositions of 5-μm-thick SiO_2_ by PECVD, followed by deposition of Cr/Ni seeding layer and patterning of photoresist where a 4-μm-thick nickel layer is electroplated inside the mold. (**b**) Pt bottom electrodes are evaporated and patterned by lift-off, then a c-axis-aligned ZnO thin film is sputter-deposited on the wafer. (**c**) Wet etching to define the contacts to the bottom electrodes using HCl to etch ZnO, then Pt top electrodes are defined via a lift-off process. (**d**) Dry etching of ZnO resonant devices and dry release by isotropic dry etching of the amorphous Si sacrificial layer.

Figure 2 presents a simplified fabrication process flow that begins with consecutive plasma-enhanced chemical vapor deposition (PECVD) depositions of a 5-μm-thick SiO_2_ isolation layer and a 1-μm-thick amorphous silicon sacrificial layer. A Cr/Ni seeding layer is then deposited, followed by patterning of an AZ P4620 photoresist mold using the first mask, where a 4-μm-thick nickel layer is electroplated inside the mold to act as the resonator body. A 200-nm-thick Pt layer is e-beam evaporated and patterned on top of the plated Ni using a lift-off process. A c-axis-aligned ZnO thin film is then deposited by RF magnetron sputtering at 300 °C (Figure 2a), which is patterned by the second mask and wet etching to define the contacts to the bottom electrodes (Figure 2b). Pt top electrodes are defined via a lift-off process using the third mask (Figure 2c). Thereafter, the fourth mask is used to generate an opening for the release process while protecting the devices. Finally, resonators are suspended by an isotropic dry etching of the amorphous Si sacrificial layer (Figure 2d) [23,24,25,26,27].

In this work, the electrolyte nickel electroplating process was performed in the nickel electroplating solution heated to ~60 °C, which was low enough to enable the post-CMOS fabrication of MEMS devices. The nickel sulfamate (Ni(SO_3_NH_2_)_2_) electrolyte process was performed to achieve low stress and high ductility. The nickel electroplating process was thoroughly characterized with a pH of 3.5 to 4.5 to reduce the roughness and avoid pitting in the plated structure. The nickel electroplating solution contains nickel sulfamate, boric acid, nickel chloride, and sodium lauryl sulfate to reduce the surface tension and improve the conductivity and brightness (surface roughness).

**Figure 3:**
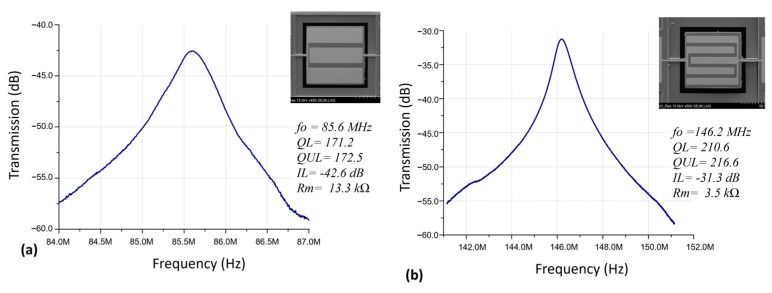
Measured frequency responses of two 82-μm-wide square plate ZnO-on-nickel resonators in its (**a**) third mode (*n* = 3) and (**b**) fifth mode (*n* = 5).

Measured frequency responses of two identically sized (82 µm wide) square plate ZnO-on-nickel resonators with varied top electrode designs are presented in Figure 3. As shown, the resonator vibrates at 85.6 MHz, which matches the resonance frequency of the third width-extensional mode (*n* = 3) based on Equation (1). Similarly, the other device operates at 146.2 MHz as shown in Figure 3b, which resonates in the fifth lateral width-extensional mode (*n* = 5). Aside from the higher resonance frequency, the fifth lateral width-extensional mode resonator exhibited a higher Q because the designed width of the top electrodes matches the mode shape (strain field) better than that of the third mode device (*n* = 3), as seen in Figure 3b. In addition, for higher order mode devices, the impedance is also reduced by a factor of *N*, where *N* = *n* for *n* equals an even number and $N=\frac{n^{2}-1}{n}$ for *n* equals an odd number [20].

**Figure 4:**
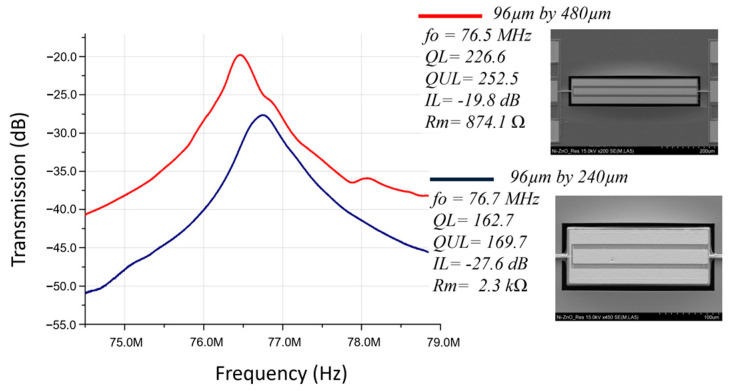
Measured frequency responses of two 96-μm-wide rectangular plate ZnO-on-nickel resonators with different IDT electrode lengths of 480 µm and 240 µm.

Aside from strategic design of the top electrode pitch size (plate width/finger number) to set the width-extensional mode frequency, the interdigital electrode length can also be varied to tune the motional impedance as seen in Figure 4. By doubling the piezoelectric thin-film transducer length, 2.63× lower motional impedance was achieved, partially due to slight Q increase.

**Figure 5:**
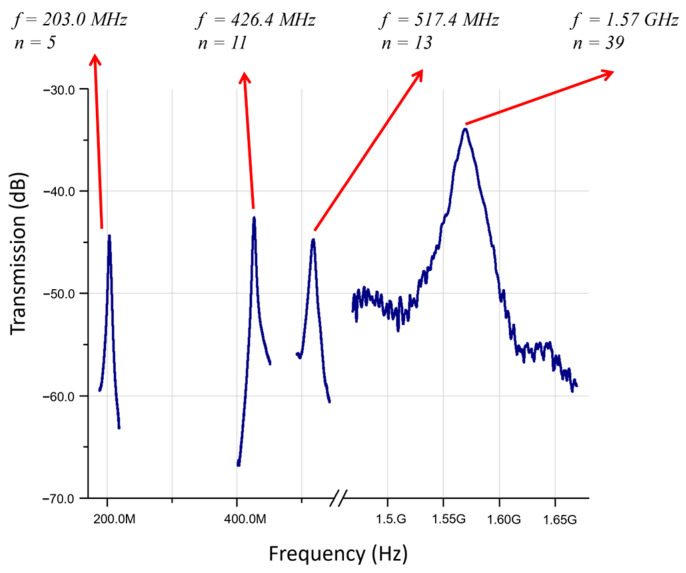
Wideband frequency responses of a 60-μm-wide square plate ZnO-on-nickel device in its fundamental (fifth width-extensional) and higher-order modes.

Besides the fundamental mode, higher order modes can also be observed in a broadband frequency spectrum of a ZnO-on-nickel resonator, as shown in Figure 5. For a square plate resonator with a width of 60 μm and five top IDT electrode fingers (*n* = 5), it operates at 203.0 MHz in the fifth width-extensional mode. Meanwhile, the higher modes of *n* = 11, 13 and 39 exhibited resonance frequencies of 426.4 MHz, 517.4 MHz and 1.57 GHz, respectively. This demonstrated that ZnO-on-nickel resonators can be designed to operate at frequencies spanning from the very high frequency (VHF) to ultra-high frequency (UHF) range.

**Figure 6:**
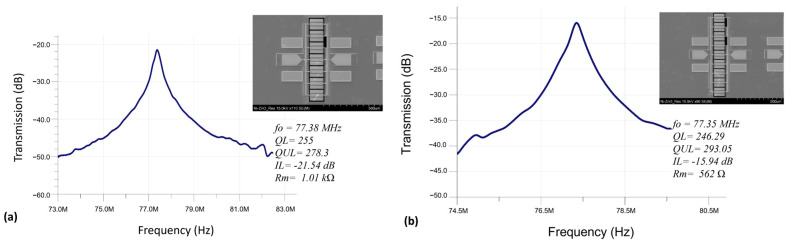
Frequency responses of ZnO-on-nickel width-extensional resonator arrays, including (**a**) a 1 × 7 resonator array; and (**b**) a 1 × 9 resonator array.

The dimensions of lateral extensional mode resonators can be shrunk to reach higher operation frequencies. As a negative effect of resonator size reduction, the electromechanical coupling coefficient (kt^2^) is lowered due to the reduced transducer area, thus resulting in a higher motional resistance. As a system-level approach, mechanically coupling several identically sized resonators into an array offers a promising solution to reduce the motional resistance while retaining the designed frequency as opposed to the electrically coupled array [20]. Under an ideal scenario, the output current can be boosted by a factor *N* that equals the total number of parallelly coupled resonators in an array by sharing the same input drive voltage. As a result, the motional resistance can be reduced by *N* times while each mechanically coupled constituent resonator device in the array resonates at the same frequency. Measured frequency responses of two resonator arrays are shown in Figure 6, and indicate a reduction of motional resistance from 1.01 kΩ to 562 Ω, when expanding the number of coupled resonators from seven to nine while retaining the same resonance frequency of 77.1 MHz. Moreover, by comparing the behavior of a single resonator in Figure 3 with the measured characteristics for coupled resonator arrays as shown in Figure 6, the effect of parallel coupling of resonators on the motional resistance can be clearly observed.

**Figure 7:**
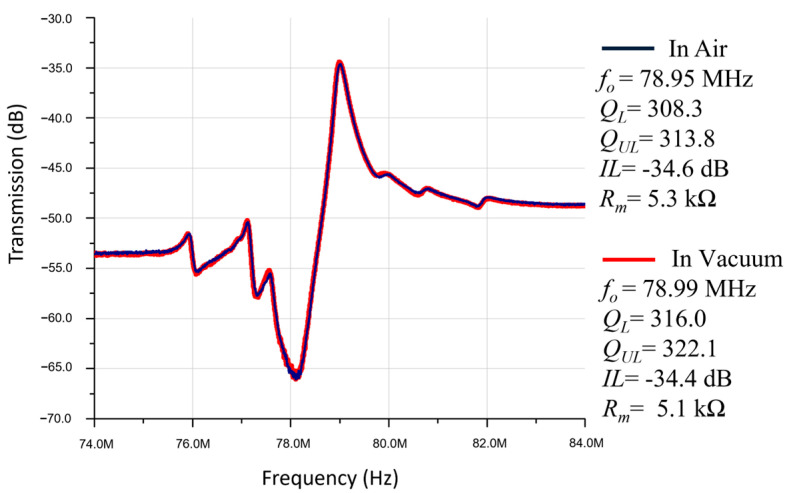
Frequency responses of a ZnO-on-nickel resonator device tested in air and vacuum. The effect of the viscous air damping seems to be insignificant.

After being wire bonded to a chip carrier, the devices were tested in a vacuum chamber. The frequency responses of a ZnO-on-nickel resonator operating in air and vacuum are presented in Figure 7. The measured Q in vacuum is only slightly better than that tested under the ambient pressure, which indicates that most of the energy losses can be ascribed to material-related dissipations, anchor losses or thermoelastic damping instead of air damping in this mode and the range of operation frequencies. The resonator in Figure 4 vibrates in the width-extensional mode, and the Q measured in-vacuum has better enhancement than the in-air measurement. In addition, the quality factor has been slightly improved due to the reduced air damping in the vacuum, which indicates that in this mode shape and range of frequency, most of the energy dissipation comes from thermoelastic loss, anchor loss, or material loss instead of air damping.

The extracted electromechanical coupling coefficient (kt^2^) factor is 4.2–4.8%, which is on par with other piezo resonators [16]. The kt^2^ factor can be calculated by the equation below:

kt^2^ = π^2^/4 × (*f*_p_ − *f*_s_)/*f*_s_ (2)

where *f*_p_ and *f*_s_ are parallel and series resonance frequencies. For the 96 µm × 480 µm rectangular plate resonator, as seen in Figure 4, the *f*_p_ and *f*_s_ are 77.8 MHz and 76.5 MHz, respectively, which corresponds to a kt^2^ of 4.2%. Similarly, the resonator array shown in Figure 6a exhibited *f*_p_ and *f*_s_ of 78.6 MHz and 77.1 MHz, respectively, which corresponds to a kt^2^ of 4.8%. Both are about half of the theoretical kt^2^ of 8.5% for ZnO-based bulk acoustic wave (BAW) resonator devices [28,29,30,31,32].

Surface roughness shows direct effects on the performance of the MEMS resonators. Most energy dissipation in the later extensional mode piezoelectric MEMS resonators occurs through surface-related losses. MEMS resonators suffer from the surface energy loss effect, and the surface stress model of the nickel can be calculated based on the elasticity of the surface stress–strain law while excluding the dissipation mechanism. By applying the idea of Zener’s “anelastic” model, the dissipative surface stress model can be written as follows [33]:

(3) $$\sigma s+т\epsilon \;\frac{\partial \sigma s}{\partial t}=\gamma +\mathrm{ES}\cdot \epsilon S+\mathrm{ES}\cdot т\sigma \cdot \frac{\partial \epsilon s}{\partial t}$$

where γ is the surface tension, ES is the elastic modulus of the surface, σs is the surface stress, ϵS is the surface strain, and тϵ and тσ represent the deformation and the dissipation relaxation times, where γ, σs, and ϵS all have the unit of force/length.

The surface of the MEMS resonator can be affected by the stress of the substrate material or the deposited metal on top of the resonator surface. The equation of the effective surface stress, σss, of the piezoelectric resonators with significant surface roughness can be written as the following [34]:

(4) $$\sigma \mathrm{ss}=\;т\epsilon \;\sqrt{{\left(\frac{\partial r}{\partial s}\right)}^{2}}\cdot (1+{\left(\frac{\partial r}{\partial s}\right)}^{2}-\frac{v}{1-v}{\left(\frac{\partial r}{\partial s}\right)}^{2})$$

where *v* is the Poisson’s ratio and r presents the roughness of the surface as a function of *s*, where the value of (*∂r*/*∂s*) is 0 when the surface of the piezoelectric resonator is totally smooth.

The electroplated nickel was carefully characterized to ensure the electroplated nickel surface was smooth enough not to impact the overall performance of the MEMS resonator. Still, the piezoelectric-on-nickel resonator degrades the device’s overall performance, such as the quality factor, due to an uncontrolled environment of the electroplating process. Accordingly, a localized annealing process can be performed to further improve the actual properties of the electroplated nickel by applying a current to the resonator body through the device’s anchors. The applied current heats the resonator to a high temperature, reaching 700–1000 °C, which removes surface contaminants and readjusts the resonator’s structural material. Above all, the electroplated nickel serves as a resonator body and a bottom electrode, which can impact the c-axis alignment of the sputtered ZnO piezoelectric layer, affecting the resonator coupling coefficient. In this work, the localized annealing processes improve the kt^2^ factor, quality factor (Q), and motional impedance by smoothing the resonator’s surface and reducing the residual stresses while improving the acoustic properties of the electroplated nickel layer. In addition, as the nickel structural material was softened, the resonator’s frequency drifted down accordingly.

**Figure 8:**
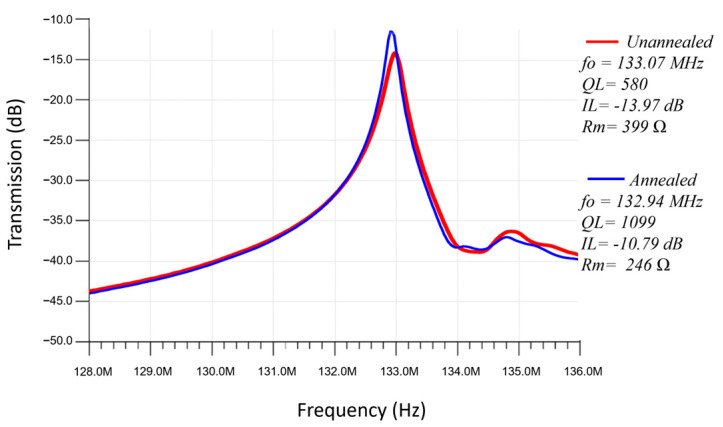
Frequency responses of ZnO-on-nickel resonator measured before and after the described localized annealing process by Joule heating effect.

Figure 8 presents the frequency responses of a rectangular-plate ZnO-on-nickel resonator tested before and after a localized annealing process was conducted. Essentially, the resonator was annealed in the ambient air for half an hour by applying a DC voltage of 1.5 V on the plated nickel microstructure via its surrounding electrical contacts to induce a Joule heating effect, which indicates a reduction of motional resistance from 399 Ω to 246 Ω. As seen in Figure 8, the resonance drifted by 0.13 MHz and the Q-factor increased by about 2× from 580 to 1099 after the localized annealing as a result of the enhanced material properties and/or surface defect removal. Increase in *Q* for capacitively transduced, comb drive, folded-beam resonators by localized annealing of electroplated nickel has been reported before [29]; our results for piezoelectric ZnO-on-nickel lateral extensional mode, rectangular-plate resonators at VHF/UHF frequencies greatly complement the prior results. Additionally, the annealed measured device in this work lowered the insertion loss by ~3 dB, resulting in a 10 dB insertion loss, which is record-breaking for MEMS electroplated nickel resonators in the literature. However, further improvement of the nickel electroplating process is needed to mitigate the higher intrinsic damping of the electroplated nickel devices. In particular, localized annealing under optimal conditions should be exploited to significantly improve the effective mechanical quality factor and lower the insertion loss for the ZnO-on-nickel resonators.

## 4. Conclusions

Lateral-extensional mode, rectangular-plate ZnO-on-nickel resonators (arrays) at VHF frequencies were explored. Resonators composed of electroplated nickel deposited at a low temperature as structural layers were fabricated, and they are well suited for back-end-of line integration. Due to the relatively high electromechanical coupling of the piezoelectric transduction, low motional impedances were obtained, which could be further reduced by optimizing the piezoelectric coefficient and area of the piezoelectric transducer layer or by parallel coupling of resonators into a resonator array. Several resonator arrays were implemented and very promising initial results were realized to validate this system-level approach for lowering motional impedance by ~50%. As seen in Table 1, the operation of the resonator in both vacuum and air was assessed, and similar Q values in the range of ~300 were observed for both. Hence, costly encapsulation may not be needed for these width-extensional mode devices. In Table 2, the measured devices of this work are benchmarked against the performance of MEMS resonators using different technologies reported in the literature. In addition, a localized thermal annealing process was also applied to reduce the residual stresses and improve the acoustic properties of the electroplated nickel microstructure, which results in considerable Q improvement from 580 to 1099, breaking the record of insertion loss in nickel-based resonators down to ~10 dB.

## Figures and Tables

**Table 1:** Measured results of ZnO-on-nickel rectangular and square resonators, illustrating the key performance parameters of the fabricated devices.

Resonator Device Structure	f_r_ (MHz)	Q	R_m_	IL (dB)
82 μm width/length, 3 IDT	85.6	172.5	13.3 kΩ	−42.6
82 μm width/length, 5 IDT	146.2	216.6	3.5 kΩ	−31.3
96 μm width, 240 μm length	76.7	169.7	2.3 kΩ	−27.6
96 μm width, 480 μm length	76.5	252.5	874.1 Ω	−19.8
1 × 7 coupled resonator array	77.1	186.6	1.1 kΩ	−21.85
1 × 9 coupled resonator array	77.1	183.5	623 Ω	−17.2
96 μm width, 120 μm length resonator tested in air	78.95	313.8	5.3 kΩ	−34.6
96 μm width, 120 μm length resonator tested in vacuum	78.99	322.1	5.1 kΩ	−34.4
60 μm width/length, 5 IDT resonator measured before localized annealing process	133.07	580	399 Ω	−13.97
60 μm width/length, 5 IDT resonator measured after localized annealing process	132.94	1099	246 Ω	−10.79

**Table 2:** MEMS nickel resonator key performance parameters in comparison to the literature.

MEMS Designs and Trabsduction Technology	f_r_ (MHz)	Q	R_m_	IL (dB)
CMOS nickel clamped-clamped beam resonator (capacitively transduced) [35]	6.62	576	-	−92
Nickel-based centrally anchored disk micro-machined resonators (capacitively transduced) [36]	15.35	66.5	-	−82
Nickel mechanically coupled flexural-mode disk resonator arrays, 9 disk resonators. (capacitively transduced) [34]	76.7	1092	5.8 kΩ	−40
Lateral-extensional mode piezoelectric ZnO-on-Nickel (piezoelectrically transduced) (this work)	132.94	1099	246 Ω	−10.76

## Data Availability

Not applicable.
